# Global and tract-specific differences between younger and older adults in DTI measures of white matter integrity

**DOI:** 10.3389/fnagi.2025.1562660

**Published:** 2025-05-30

**Authors:** Stephanie Matijevic, Lee Ryan

**Affiliations:** ^1^Department of Psychology, University of Arizona, Tucson, AZ, United States; ^2^Evelyn F. McKnight Brain Institute, University of Arizona, Tucson, AZ, United States

**Keywords:** aging, diffusion tensor imaging, white matter, neuroimaging, MRI

## Abstract

Prior research utilizing diffusion tensor imaging (DTI) to examine cerebral white matter microstructural integrity among adults has established that increasing age is associated with poorer white matter health. While age effects on DTI measures of white matter integrity have been shown to vary in strength across different white matter tracts, tract-specific effects may be secondary to a global impact of age on white matter health. Furthermore, this global age effect could result in “homogenizing” increases in shared variance across tracts. The present study compared DTI measures in 36 white matter tracts between 71 younger adults (ages 18–37) and 129 older adults (ages 52–82), to (1) determine whether shared variance across white matter tracts increases with age, and (2) examine tract-specific variability in age-related alterations to white matter integrity. Diffusion weighted images were processed using probabilistic tractography in order to reconstruct callosal, association, and radiation tracts, from which average measures of fractional anisotropy (FA), mean diffusivity (MD), radial diffusivity (RD) and axial diffusivity (AD) were derived. In comparing inter-tract correlation matrices for each DTI measure between age groups, we found stronger inter-tract correlations for older adults relative to younger adults overall. Additionally, general factors for FA, RD and AD, derived from separate factor analyses, accounted for greater proportions of shared variance across tracts among older adults compared to younger adults. For MD, however, the amount of shared variance captured by the general factor was similar between age groups. Older adults exhibited lower FA and higher MD and RD values compared to younger adults for the majority of tracts examined, although the strength of the age effect differed across tracts. Age group differences in AD were more variable. The present findings provide support for the notion that aging exerts a global, homogenizing impact on white matter integrity, alongside tract-specific age effects.

## 1 Introduction

The integrity of cerebral white matter is a key factor in the successful execution of a wide variety of cognitive functions, including executive functions (Ribeiro et al., 2024; Ryan and Walther, 2014) and memory (Jiménez-Balado et al., 2022; Read et al., 2023). White matter integrity tends to be compromised with increasing age in multiple ways including myelin sheath splitting, accumulation of cytoplasm between lamellae, axonal shrinkage, inflammatory damage, and gliosis, as observed in animal models of aging (see Peters, 2009; Groh and Simons, 2024 for reviews). In humans, diffusion tensor imaging (DTI) has been used extensively to characterize the impact of aging on white matter microstructural integrity. Prior studies have reported largely consistent findings of age-related alterations in DTI metrics of white matter integrity. Fractional anisotropy (FA), a measure of the directionality of diffusion that serves as an index of white matter tract coherence, has been shown to decrease with age (Lebel et al., 2012; Storsve et al., 2016; Cox et al., 2016; Schilling et al., 2022), while mean diffusivity (MD), a measure of the overall magnitude of diffusion, increases with age (Lebel et al., 2012; Storsve et al., 2016; Cox et al., 2016; Schilling et al., 2022). These age-associated alterations to FA and MD are interpreted as indicators of white matter damage, likely driven by a combination of the various factors that impact white matter health in aging. Age-related increases have also been observed for radial diffusivity (RD; Bennett et al., 2010; Brickman et al., 2012; Schilling et al., 2022) and axial diffusivity (AD; Bennett et al., 2010; Schilling et al., 2022), both direction-specific measures of the magnitude of diffusion. Increases in RD and AD may reflect demyelination and axonal degeneration, respectively (Song et al., 2002; Song et al., 2003; Song et al., 2005), although the specificity of these measures might be reduced in the presence of mixed demyelinating and degenerating white matter pathology (Winklewski et al., 2018).

Prior research has suggested that age-related alterations to DTI metrics are variable across cerebral white matter, with some tracts showing greater vulnerability to aging than others. For instance, stronger age effects have been reported for association tracts compared to sensorimotor tracts (de Groot et al., 2015; Tseng et al., 2021). Differences in microstructural profiles may render some tracts more sensitive to aging than others. As an example, in the case of the corpus callosum, regional differences in myelin density may explain why callosal segments vary in their relationships with age. The anterior genu has a greater proportion of unmyelinated and thinly myelinated fibers than posterior regions like the splenium, and also shows greater DTI metric alterations with age than the splenium (Aboitiz et al., 1992; McLaughlin et al., 2007; Pietrasik et al., 2020). Additionally, spatial location may also determine tract sensitivity to age. White matter within “watershed” areas – cerebral artery boundary zones that are particularly vulnerable to reductions in cerebral perfusion – may sustain more damage due to age-related hypoperfusion than other areas. This idea is supported by findings from older adults that MRI white matter signal abnormalities are abundant in watershed areas like periventricular white matter (Lindemer et al., 2017), and that reduced cerebral blood flow to periventricular areas is predictive of white matter lesion expansion (Promjunyakul et al., 2018).

While age effects on white matter integrity may not be uniform in strength across tracts, age may nonetheless have a “global” impact on white matter integrity, as indicated by evidence that age impacts the majority of tracts to some degree (Cox et al., 2016; Schilling et al., 2022). Age-related white matter microstructural alterations, like demyelination and axonal loss, and the mechanisms that contribute to such alterations, like cerebrovascular dysfunction, presumably do not target specific tracts, but instead affect white matter across the cerebrum. In line with this, prior studies have demonstrated that a general factor can be extracted that accounts for substantial variance across white matter tract DTI measures among middle-aged and older adults (Cox et al., 2016; Gustavson et al., 2019; Penke et al., 2010). For example, in a sample of 3,513 adults ages 44–77, Cox et al. (2016) found that a general factor accounted for 41.4% of the variance in FA values across multiple tracts, including association tracts, projection tracts, and thalamic radiations. Similarly, Penke et al. (2010) extracted a general factor accounting for 40.28% of the variance in FA values across callosal and frontal tracts, among 132 older adults ages 71–72. Among 390 middle-aged adult male twins ages 56–66, Gustavson et al. (2019) found a single factor accounting for 50.3% of the variance in FA values across 11 tracts, including association and callosal tracts.

It is important to note that the impact of age on white matter integrity need not be characterized as exclusively either global or tract-specific. As evidence of this, while Cox et al. (2016) reported a single factor accounting for substantial variance in FA across multiple tracts, they also noted tract-specific variability in the strength of the association between age and FA. Specifically, they found stronger age relationships for thalamic radiations and association tracts compared to projection tracts. Similarly, we previously reported that increasing age was associated with reduced FA in select tracts among older adults ages 54–92 at baseline, even after accounting for whole brain white matter FA (Matijevic and Ryan, 2021). The co-occurrence of global and tract-specific effects of age on white matter integrity highlights the importance of accounting for global DTI measures when exploring potential relationships between specific white matter tracts and variables like cognitive function. For example, in a longitudinal study of healthy older adults, Rabin et al. (2019a) observed that lower global FA at baseline predicted declines in processing speed and episodic memory performance, whereas tract-specific FA measures did not predict cognitive change after controlling for global FA. In this same cohort, however, lower fornix FA interacted with elevated *β*-amyloid burden to predict accelerated episodic memory decline, an effect that persisted even after controlling for global FA (Rabin et al., 2019b). β-amyloid burden did not interact with FA in other tracts or with global FA to predict cognitive changes, suggesting that this relationship was unique to the fornix. Taken together, these studies illustrate how evidence for tract-specific relationships with cognition can be strengthened when global white matter measures are taken into account.

The benefits of controlling for global white matter in analyses of tract-specific DTI measures may be heightened when working with data from older adults or samples with wide age ranges. Lövdén et al. (2013) hypothesized that a strong global effect of age on white matter integrity could in turn result in “homogenization,” such that shared variance across tracts is increased among older adults. Lövdén et al. (2013) tested this hypothesis in a sample of 260 older adults, comparing correlations among DTI measures of multiple tracts between a “younger” group of older adults (ages 60–72) and an “older” group of older adults (ages 78–87). They found that FA and MD inter-tract correlations did not generally differ between the age groups. In contrast, Cox et al. (2016) found that the proportion of total variance shared across tract FA and MD measures increased with age, among adults ages 44–77. The discrepancy between these two findings may lie in the sample age ranges; the homogenizing effect of increasing age on white matter tract DTI measures may be more apparent when comparing older adults to those in younger age brackets. To the best of our knowledge, no such comparison between older adults and younger adults (ages 20s and 30s) has been conducted.

The purpose of the present study was two-fold. First, we investigated whether increasing age has a homogenizing effect on DTI measures of white matter microstructural integrity across tracts by examining four metrics – FA, MD, RD, and AD - in 36 tracts for 71 younger adults (ages 18–37) and 129 older adults (ages 52–82). Specifically, we compared inter-tract correlation matrices and general white matter factors (derived via factor analysis) between age groups, predicting that shared variance across tract DTI measures would be higher among older adults relative to younger adults in line with the hypothesis of age-related “homogenization.” Second, we evaluated age group differences in DTI metric values to determine whether some tracts are more sensitive than others to age-related changes in white matter integrity.

## 2 Materials and methods

### 2.1 Participants

The present sample consisted of 129 older adults, ages 52–82, and 71 younger adults, ages 18–37 (see Table 1 for demographics). Younger and older adults did not differ in years of education [*t*(139.48) = 0.62, *p* = 0.533]. Participants were screened and excluded for neurological disorders, drug/alcohol abuse, psychiatric disorders, traumatic brain injury, and MRI contraindications. Older adult participants scored within normal limits on the Mini Mental Status Exam (Folstein et al., 1975). Written, informed consent was obtained in accordance with the guidelines set by the University of Arizona’s institutional review board.

**Table 1:** Demographics for the younger adults (YA) and older adults (OA) in the present study sample.

	YA (*N* = 71)	OA (*N* = 129)
Age		
Mean (SD)	26.2 (4.01)	67.9 (5.88)
Min, Max	18, 37	52, 82
Sex (*N*, %)		
Female	43 (60.6%)	90 (69.8%)
Male	28 (39.4%)	39 (30.2%)
Education		
Mean (SD)	17.0 (2.17)	16.8 (2.08)
Min, Max	12, 23	12, 22

### 2.2 MRI acquisition

All participants were scanned on a 3T Siemens Skyra. A 3D high resolution T1 weighted image was collected with the following parameters: voxel size = 1.1 × 1.1 × 1.0 mm, TR = 2,300 ms, TE = 2.95 ms, FOV = 270 mm. Diffusion weighted images were collected in 64 directions (along with 6 non-diffusion weighted images) with the following parameters: b-value = 1,000 s/mm^2^, voxel size = 2.0 mm^3^, TR = 10,000 ms, TE = 80 ms, FOV = 254 mm, slice thickness = 2 mm, GRAPPA acceleration factor = 2. The diffusion weighted imaging sequence varied slightly across two sets of participants – for 137 participants, 60 slices were collected per volume, while for 63 other participants, 74 slices were collected per volume. There was no significant difference in the proportion of younger and older adults who were scanned with one diffusion weighted imaging sequence versus another [*χ*^2^(1) = 3.809, *p* > 0.05].

### 2.3 Image processing

The high resolution T1 weighted images were processed with Freesurfer 7.3.2’s *recon-all* and thalamic nuclei segmentation pipelines (Fischl, 2012; Iglesias et al., 2018) in order to produce subcortical segmentations and cortical parcellations. The outputs of these pipelines were then passed, along with diffusion weighted images, to Freesurfer’s TRACULA (Yendiki et al., 2011; Maffei et al., 2021). Within this pipeline, diffusion weighted images were corrected for motion and eddy current distortions with FSL’s *eddy* tool (Andersson and Sotiropoulos, 2016), and each participant’s first b0 image was registered to their respective T1 image using *bbregister* (Greve and Fischl, 2009). Tensor fitting was next performed using FSL’s *dtifit*, and the resulting FA maps were non-linearly registered to the Human Connectome Project FA template via Advanced Normalization Tools (Avants et al., 2011). Intra- and inter-participant transformations were combined to allow normalization of participants’ subcortical segmentations and cortical parcellations to template space. The normalized subcortical segmentations and cortical parcellations were used, in combination with a manually annotated set of tracts from a training dataset in template space, to determine probable tract trajectories for each participant based on their individual anatomy.

**Figure 1:**
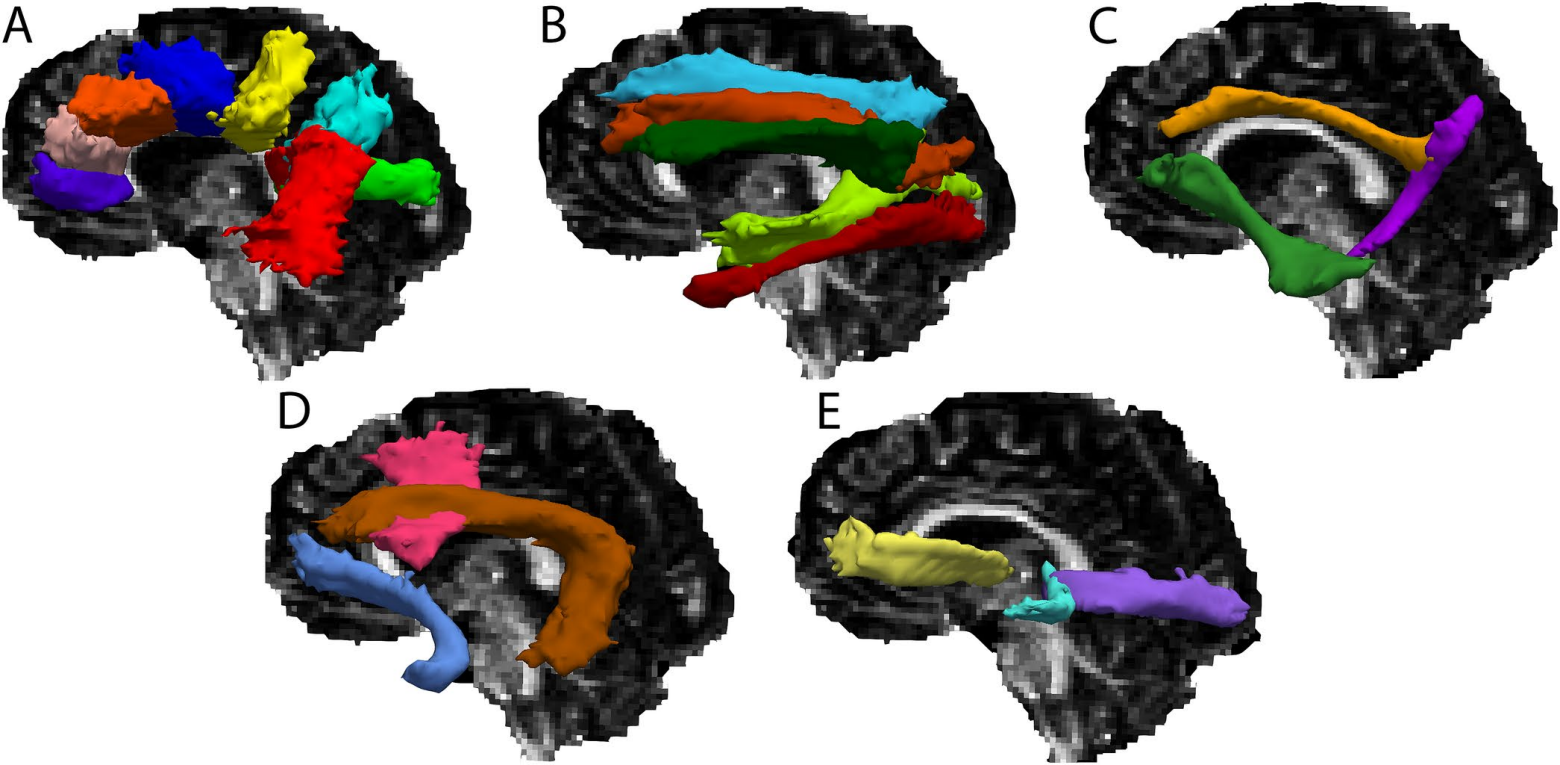
Cerebral white matter tracts for a single participant overlaid on the participant’s FA map. **(A)** Rostrum of the corpus callosum (purple), genu of the corpus callosum (pink), body of the corpus callosum – prefrontal (orange), body of the corpus callosum – premotor (dark blue), body of the corpus callosum – central (yellow), body of the corpus callosum – parietal (light blue), body of the corpus callosum – temporal (red), splenium of the corpus callosum (green). **(B)** Superior longitudinal fasciculus – I (blue), superior longitudinal fasciculus – II (orange), superior longitudinal fasciculus – III (dark green), middle longitudinal fasciculus (light green), inferior longitudinal fasciculus (red). **(C)** Dorsal cingulum bundle (gold), ventral cingulum bundle (purple), extreme capsule (green). **(D)** Frontal aslant tract (pink), arcuate fasciculus (brown), uncincate fasciculus (blue). **(E)** Anterior thalamic radiation (yellow), acoustic radiation (blue), optic radiation (purple).

For the present study, a total of 36 cerebral white matter tracts were reconstructed through probabilistic tractography via TRACULA, for each participant (see Table 2 for a list of tracts and Figure 1 for a visual depiction of the tracts). First, a ball-and-stick model was fit at each voxel within the diffusion weighted imaging data, using FSL’s *bedpostx* (Behrens et al., 2007). Then, using a Monte Carlo Markov chain algorithm, probabilistic distributions were computed for each tract by combining diffusion orientation information at each voxel with the anatomically-derived estimates of probable tract trajectories. The resulting tract probability distributions were thresholded such that only voxels with a probability >20% of the tract’s maximum probability value remained (Yendiki et al., 2011). For each thresholded tract probability distribution, we calculated average FA, MD, RD and AD, weighted by the probability value at each voxel. All tracts for all participants were visually assessed to ensure that reconstruction was completed successfully.

**Table 2:** List of the 36 cerebral white matter tracts reconstructed for the present study.

Corpus callosum tracts	Thalamic radiations
Rostrum (CC.R)	Left anterior thalamic radiation (L.ATR)
Genu (CC.G)	Right anterior thalamic radiation (R.ATR)
Body – prefrontal (CC.Bpf)	Left acoustic radiation (L.AR)
Body – premotor (CC.Bpm)	Right acoustic radiation (R.AR)
Body – central (CC.Bc)	Left optic radiation (L.OR)
Body – parietal (CC.Bp)	Right optic radiation (R.OR)
Body – temporal (CC.Bt)	
Splenium (CC.S)	
Association tracts	
Left arcuate fasciculus (L.AF)	Left middle longitudinal fasciculus (L.MLF)
Right arcuate fasciculus (R.AF)	Right middle longitudinal fasciculus (R.MLF)
Left dorsal cingulum bundle (L.CBD)	Left superior longitudinal fasciculus – I (L.SLFI)
Right dorsal cingulum bundle (R.CBD)	Right superior longitudinal fasciculus – I (R.SLFI)
Left ventral cingulum bundle (L.CBV)	Left superior longitudinal fasciculus – II (L.SLFII)
Right ventral cingulum bundle (R.CBV)	Right superior longitudinal fasciculus – II (R.SLFII)
Left extreme capsule (L.EMC)	Left superior longitudinal fasciculus – III (L.SLFIII)
Right extreme capsule (R.EMC)	Right superior longitudinal fasciculus – III (R.SLFIII)
Left frontal aslant tract (L.FAT)	Left uncincate fasciculus (L.UF)
Right frontal aslant tract (R.FAT)	Right uncincate fasciculus (R.UF)
Left inferior longitudinal fasciculus (L.ILF)	
Right inferior longitudinal fasciculus (R.ILF)	

### 2.4 Statistical analyses

In order to test our hypothesis of age-related “homogenization” of white matter DTI measures, several analyses were conducted. First, per DTI metric (FA, MD, RD, and AD), matrices of Pearson correlations between tracts were computed for younger adults and older adults separately. Age group differences between the correlation matrices were assessed via the Jennrich test (Jennrich, 1970), which employs a *χ*^2^ method of testing the equality of independent correlation matrices. This omnibus test was followed up with Fisher’s *z*-tests (uncorrected *p* < 0.05) to compare individual inter-tract correlations between younger and older adults. We additionally explored the “homogenization” hypothesis by conducting factor analyses of the tract measures, one per DTI metric and age group. Each model was designed to extract a single, unrotated factor through maximum-likelihood factor analysis. Lastly, regression models were set up to test for age group differences in the tract DTI metrics, with age group (younger adult, older adult) entered as a predictor while sex and diffusion weighted imaging sequence type served as covariates. An FDR corrected *p* < 0.05 was applied across all regressions.

## 3 Results

### 3.1 Age group differences in inter-tract correlations

Across all four DTI metrics, Jennrich tests revealed that tract correlation matrices differed between the age groups. For FA, RD, and AD, inter-tract correlations were generally higher for older adults compared to younger adults, as indicated by Fisher’s *z*-tests. The specific results of these analyses are listed below.

**Figure 2:**
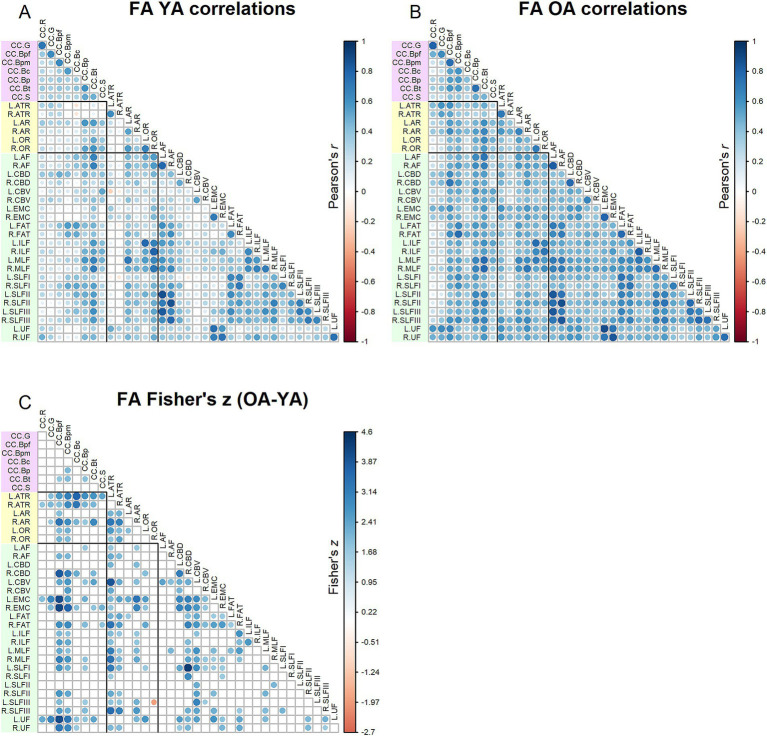
Top - matrices showing **(A)** tract FA correlations for younger adults and **(B)** tract correlations for older adults, in which correlation coefficient strength is represented by circle size and color (see legends). Bottom – matrix showing **(C)** results of Fisher’s *z*-tests on tract FA correlations, in which only significant (*p* < 0.05) results are shown. Positive Fisher’s *z* values (blue) denote correlations where older adults had stronger correlation coefficients than younger adults, while negative Fisher’s *z* values (red) denote correlations where younger adults had stronger correlation coefficients than older adults. The size and color of the circles represent the magnitude of the Fisher’s *z* statistic (see legend).

Correlation matrices for FA values are shown in Figure 2. Results of the Jennrich test indicated that the matrices differed between older and younger adults [*χ*2(630) = 3085.08, *p* < 0.001]. The Fisher’s *z*-tests (Figure 2) revealed 225 correlations in which age groups differed. For almost all the inter-tract correlations showing significant age group differences, older adults had stronger correlations compared to younger adults. Higher inter-tract correlations were especially notable for the body of the corpus callosum, the thalamic radiations and cingulum bundles.

**Figure 3:**
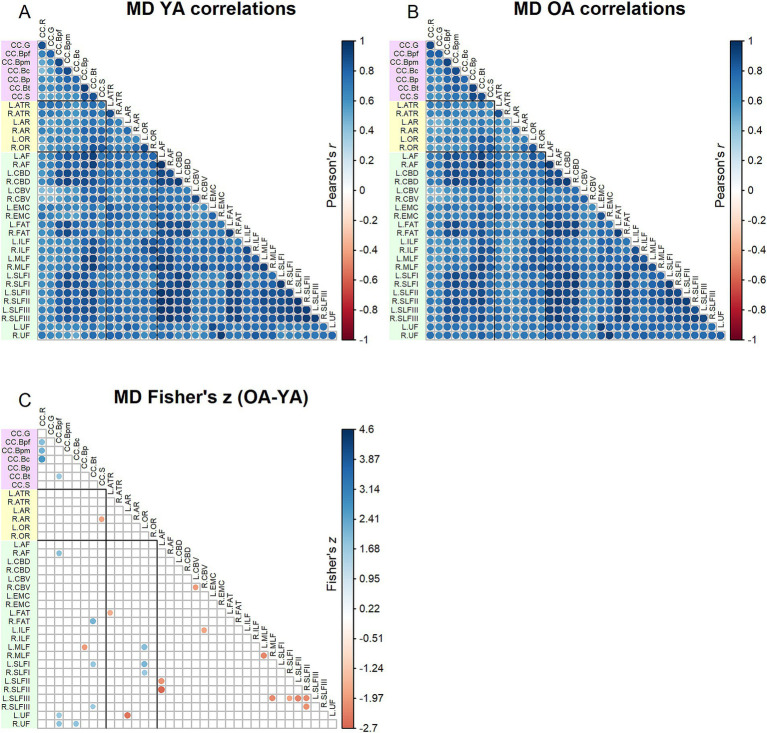
Top - matrices showing **(A)** tract MD correlations for younger adults and **(B)** tract correlations for older adults, in which correlation coefficient strength is represented by circle size and color (see legends). Bottom – matrix showing **(C)** results of Fisher’s *z*-tests on tract MD correlations, in which only significant (*p* < 0.05) results are shown. Positive Fisher’s *z* values (blue) denote correlations where older adults had stronger correlation coefficients than younger adults, while negative Fisher’s *z* values (red) denote correlations where younger adults had stronger correlation coefficients than older adults. The size and color of the circles represent the magnitude of the Fisher’s *z* statistic (see legend).

Correlation matrices for MD values are shown in Figure 3. Comparing Figures 2, 3, MD inter-tract correlations overall appeared higher (for both younger and older adults) relative to FA. The Jennrich test indicated that MD matrices differed between older and younger adults [*χ*^2^(630) = 4129.64, *p* < 0.001]. The Fisher’s *z*-tests (Figure 3) revealed 28 correlations in which younger and older adults differed, with older adults showing both stronger and weaker correlations compared to younger adults.

**Figure 4:**
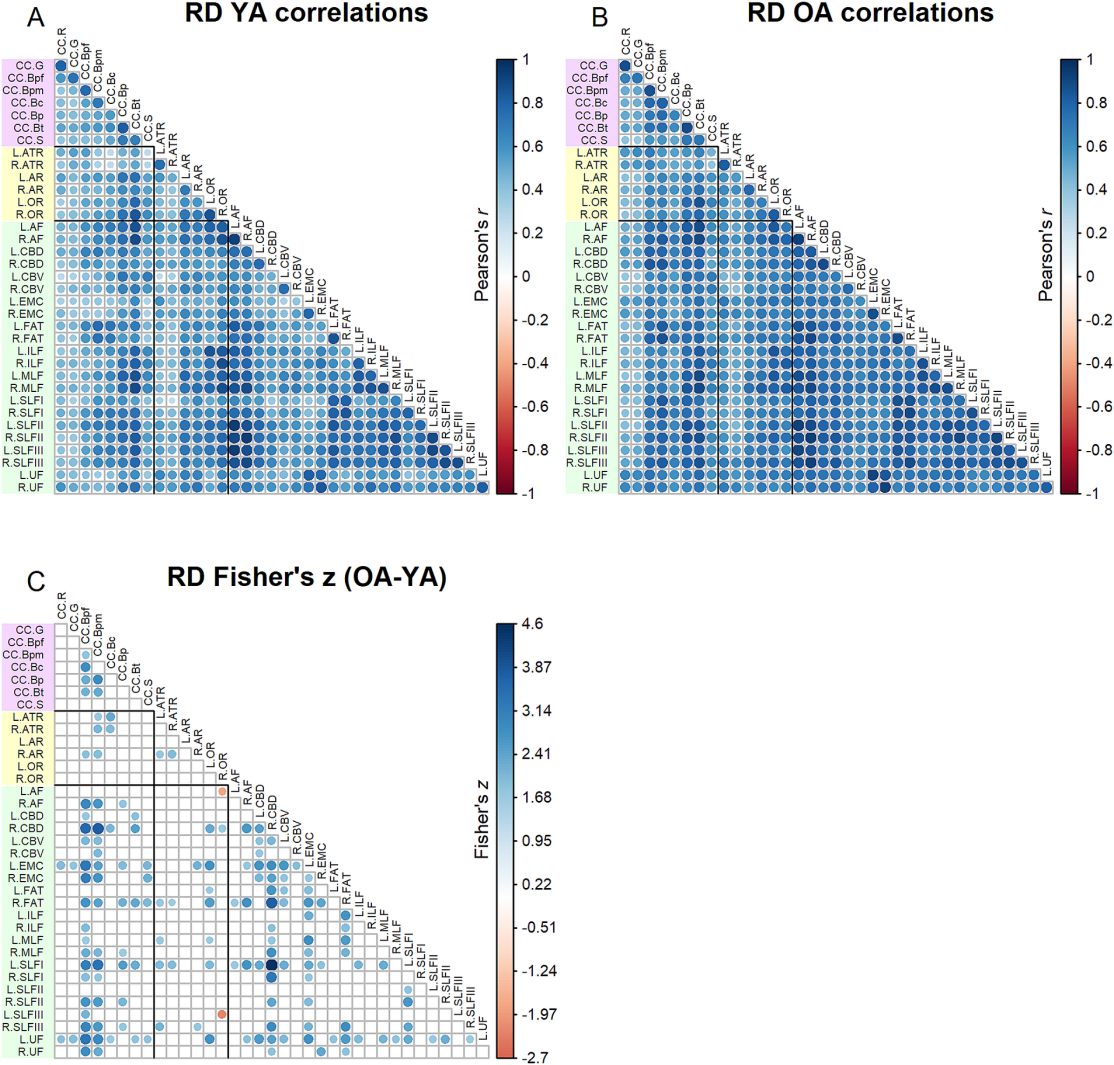
Top - matrices showing **(A)** tract RD correlations for younger adults and **(B)** tract correlations for older adults, in which correlation coefficient strength is represented by circle size and color (see legends). Bottom – matrix showing **(C)** results of Fisher’s *z*-tests on tract RD correlations, in which only significant (*p* < 0.05) results are shown. Positive Fisher’s *z* values (blue) denote correlations where older adults had stronger correlation coefficients than younger adults, while negative Fisher’s *z* values (red) denote correlations where younger adults had stronger correlation coefficients than older adults. The size and color of the circles represent the magnitude of the Fisher’s *z* statistic (see legend).

Correlation matrices for RD values are shown in Figure 4. Results of the Jennrich test indicated that the matrices differed between older and younger adults [*χ*^2^(630) = 4932.59, *p* < 0.001]. Similar to the results for FA, the Fisher’s *z*-tests (Figure 4) for RD inter-tract correlations revealed numerous correlations (154) in which younger adults and older adults differed, with older adults primarily showing stronger correlations than younger adults. Correlations among older adults were particularly higher for the body of the corpus callosum, right cingulum bundle, left extreme capsule and left uncinate fasciculus.

**Figure 5:**
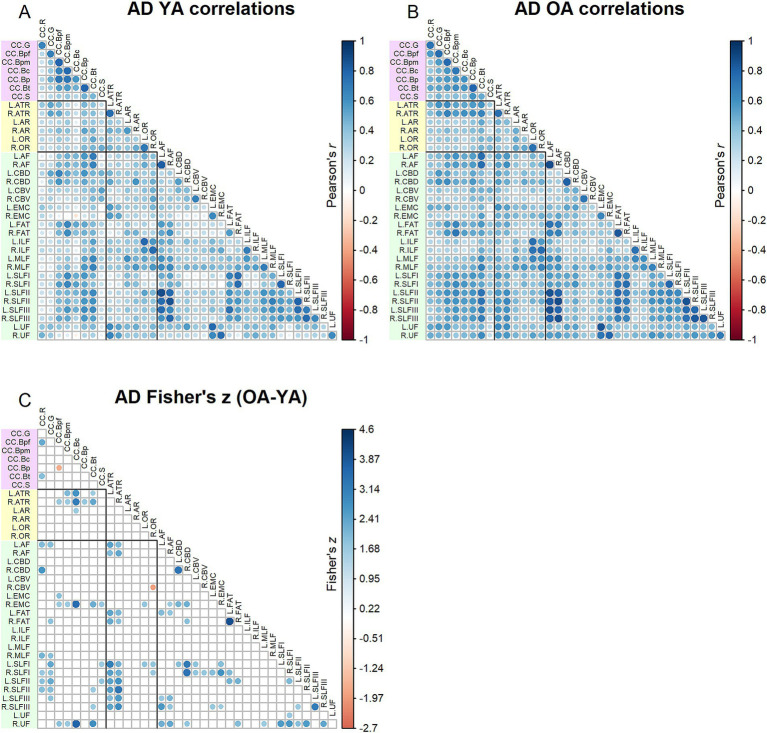
Top - matrices showing **(A)** tract AD correlations for younger adults and **(B)** tract correlations for older adults, in which correlation coefficient strength is represented by circle size and color (see legends). Bottom – matrix showing **(C)** results of Fisher’s *z*-tests on tract AD correlations, in which only significant (*p* < 0.05) results are shown. Positive Fisher’s *z* values (blue) denote correlations where older adults had stronger correlation coefficients than younger adults, while negative Fisher’s *z* values (red) denote correlations where younger adults had stronger correlation coefficients than older adults. The size and color of the circles represent the magnitude of the Fisher’s *z* statistic (see legend).

Correlation matrices for AD values are shown in Figure 5. Results of the Jennrich test indicated that the matrices differed between older and younger adults [*χ*^2^(630) = 2422.70, *p* < 0.001]. The Fisher’s *z*-tests (Figure 5) revealed 105 inter-tract correlations in which younger adults and older adults differed, with older adults mostly showing stronger correlations than younger adults. Higher correlations among older adults were especially prominent for the anterior thalamic radiations and superior longitudinal fasciculi.

### 3.2 Factor analyses of tract DTI measures

Through factor analysis, we extracted a single or “general” factor capturing the variance common across tract measures for each DTI metric, among younger adults and older adults separately. Table 3 lists the variance accounted for in each model. The amount of shared variance explained by the general factor differed across DTI metrics, with higher values for MD and RD and lower values for FA and AD overall. For all four DTI metrics, the variance accounted for by the general factor was higher for older adults compared to younger adults, with differences ranging from 0.7% for MD to 13.4% for FA. Across all factor analyses, tract measures loaded onto the factors in a positive direction. Most factor loadings were at least of moderate strength (> 0.3), with the exception of three FA tract measures among younger adults – the left anterior thalamic radiation, right anterior thalamic radiation and left ventral cingulum bundle (see Supplementary Table 1 for the factor loadings from each factor analysis performed).

**Table 3:** The percentage of variance explained by the factors extracted in the factor analyses, per age group and DTI metric.

	FA	MD	RD	AD
Younger adults	35.1	74.6	60.9	40
Older adults	48.5	75.3	69.2	47.7
Difference between age groups	13.4	0.7	8.3	7.7

The last row shows the difference in the percentage of variance explained across older adults and younger adults.

### 3.3 Age group differences in tract DTI measures

**Figure 6:**
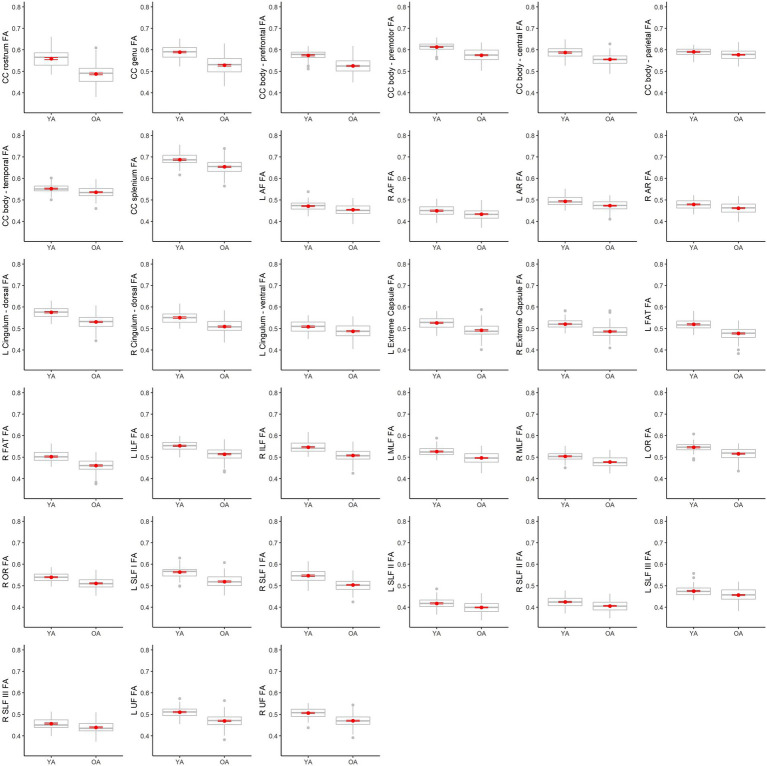
Boxplots showing differences in tract FA values between younger adults (YA) and older adults (OA). Red dots represent the means, red error bars represent the standard error (± 1 SE), and gray dots are outliers (values 1.5 times the interquartile range over the third quartile or under the first quartile).

**Figure 7:**
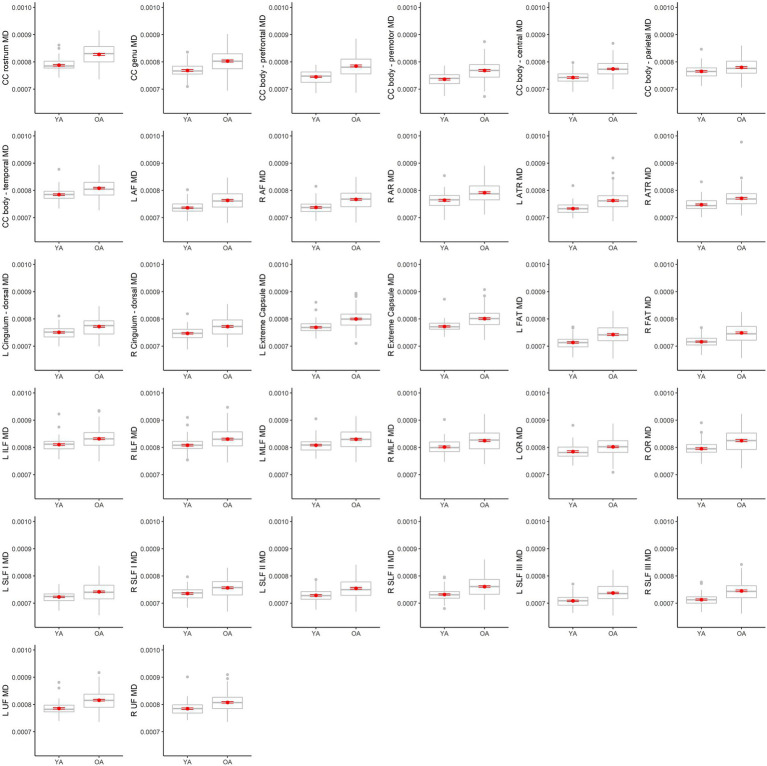
Boxplots showing differences in tract MD values between younger adults (YA) and older adults (OA). Red dots represent the means, red error bars represent the standard error (± 1 SE), and gray dots are outliers (values 1.5 times the interquartile range over the third quartile or under the first quartile).

**Figure 8:**
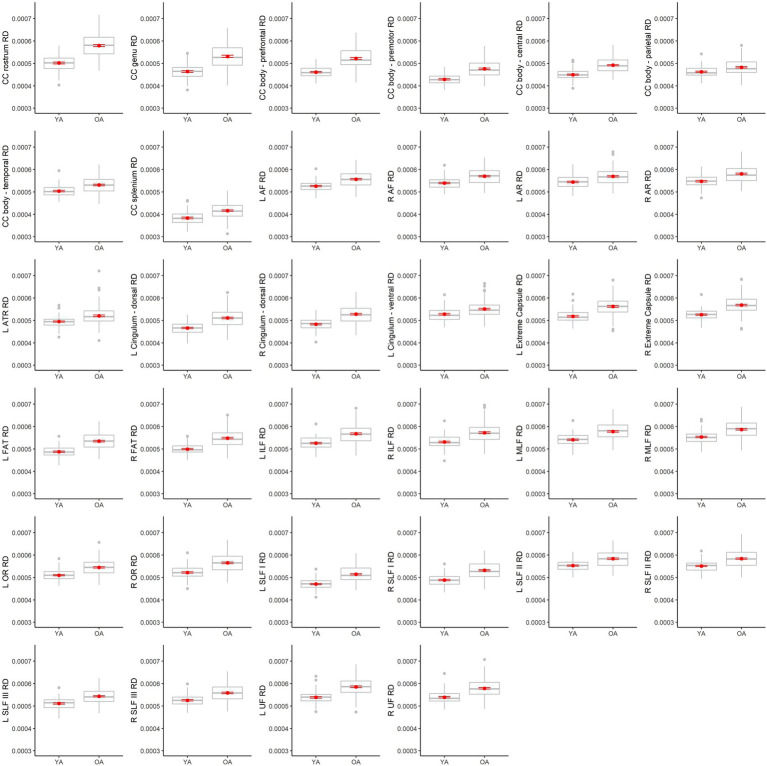
Boxplots showing differences in tract RD values between younger adults (YA) and older adults (OA). Red dots represent the means, red error bars represent the standard error (± 1 SE), and gray dots are outliers (values 1.5 times the interquartile range over the third quartile or under the first quartile).

Table 4 lists results from the regressions testing for age group differences in tract DTI metrics. In the majority of tracts, older adults exhibited lower FA, and higher MD and RD, relative to younger adults. Specifically, older adults had significantly lower FA values than younger adults for 33 out of the 36 assessed tracts (Figure 6). For tracts showing significant age differences in FA, model *R^2^* values ranged substantially, from 7% (ex. parietal body of the corpus callosum) to 43% (ex. rostrum of the corpus callosum). For 32 tracts, older adults exhibited higher MD values than younger adults (Figure 7), with model *R^2^* values ranging from 6% (ex. left optic radiation) to 27% (ex. central body of the corpus callosum). For 34 tracts, RD values were higher for older adults compared to younger adults (Figure 8). Like FA, model *R^2^* values ranged substantially for RD values, from 8% (ex. parietal body of the corpus callosum) to 41% (ex. rostrum of the corpus callosum). Supplementary Figures 1–4 display plots of age group differences in all tract DTI measures, both significant and non-significant.

**Table 4:** Results of regressions testing for age group differences in tract DTI metrics.

Tract	FA		MD		RD		AD	
	*β*	Adj. *R^2^*	*β*	Adj. *R^2^*	*β*	Adj. *R^2^*	*β*	Adj. *R^2^*
CC.R	0.59***	0.43	−0.47***	0.23	−0.6***	0.41	0.27*	0.11
CC.G	0.55***	0.39	−0.41***	0.16	−0.52***	0.33	0.22	0.16
CC.Bpf	0.62***	0.40	−0.5***	0.24	−0.61***	0.35	−0.004	0.04
CC.Bpm	0.59***	0.33	−0.48***	0.22	−0.58***	0.32	−0.07	0.02
CC.Bc	0.53***	0.27	−0.53***	0.27	−0.6***	0.34	−0.13	0.05
CC.Bp	0.28**	0.07	−0.26*	0.08	−0.31**	0.08	−0.09	0.09
CC.Bt	0.31**	0.10	−0.38***	0.14	−0.38***	0.13	−0.23	0.11
CC.S	0.48***	0.23	−0.17	0.04	−0.45***	0.21	0.3**	0.08
L.AF	0.31**	0.10	−0.43***	0.17	−0.43***	0.17	−0.29**	0.09
R.AF	0.29**	0.07	−0.45***	0.18	−0.43***	0.16	−0.35***	0.12
L.AR	0.41***	0.15	−0.23	0.04	−0.37***	0.12	0.09	< 0.01
R.AR	0.32***	0.09	−0.38***	0.13	−0.42***	0.16	−0.21	0.04
L.ATR	0.09	0.05	−0.42***	0.18	−0.29**	0.10	−0.44***	0.19
R.ATR	0.03	0.02	−0.35***	0.12	−0.24	0.06	−0.34***	0.13
L.CBD	0.58***	0.34	−0.36***	0.12	−0.53***	0.27	0.23	0.12
R.CBD	0.55***	0.30	−0.4***	0.15	−0.54***	0.27	0.12	0.05
L.CBV	0.33***	0.09	−0.19	0.05	−0.33***	0.11	0.1	0.02
R.CBV	0.25	0.05	−0.09	0.05	−0.24	0.06	0.15	0.07
L.EMC	0.49***	0.25	−0.45***	0.19	−0.5***	0.24	−0.08	0.01
R.EMC	0.53***	0.26	−0.43***	0.19	−0.52***	0.26	0.004	0.02
L.FAT	0.6***	0.35	−0.43***	0.18	−0.57***	0.31	0.08	< 0.01
R.FAT	0.59***	0.33	−0.48***	0.21	−0.59***	0.32	0.003	< 0.01
L.ILF	0.58***	0.32	−0.31**	0.09	−0.5***	0.23	0.19	0.03
R.ILF	0.59***	0.34	−0.3**	0.09	−0.48***	0.23	0.16	0.01
L.MLF	0.52***	0.25	−0.32***	0.09	−0.47***	0.20	0.11	< 0.01
R.MLF	0.48***	0.22	−0.34***	0.11	−0.43***	0.17	−0.05	0.02
L.OR	0.53***	0.26	−0.27*	0.06	−0.48***	0.22	0.17	0.01
R.OR	0.52***	0.26	−0.38***	0.13	−0.52***	0.25	−0.002	−0.01
L.SLFI	0.61***	0.36	−0.32***	0.10	−0.56***	0.30	0.28**	0.12
R.SLFI	0.62***	0.36	−0.35***	0.12	−0.56***	0.30	0.23	0.08
L.SLFII	0.34***	0.12	−0.4***	0.14	−0.42***	0.16	−0.23	0.04
R.SLFII	0.36***	0.11	−0.44***	0.17	−0.45***	0.18	−0.29**	0.07
L.SLFIII	0.33***	0.11	−0.46***	0.20	−0.46***	0.20	−0.33***	0.10
R.SLFIII	0.31**	0.09	−0.47***	0.20	−0.44***	0.18	−0.39***	0.15
L.UF	0.55***	0.35	−0.43***	0.17	−0.53***	0.27	0.01	0.06
R.UF	0.54***	0.29	−0.35***	0.13	−0.48***	0.23	0.14	0.02

Age effect *β*’s are shown next to the adjusted *R^²^* for the model. FDR-corrected *p*-values are represented as follows: *** *p* < 0.001, ** *p* < 0.01, * *p* < 0.05.

**Figure 9:**
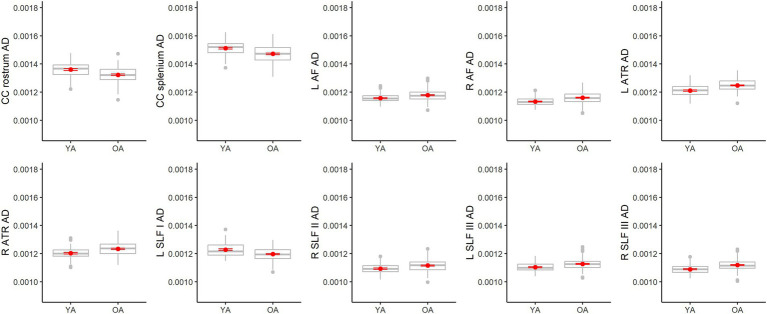
Boxplots showing differences in tract AD values between younger adults (YA) and older adults (OA). Red dots represent the means, red error bars represent the standard error (± 1 SE), and gray dots are outliers (values 1.5 times the interquartile range over the third quartile or under the first quartile).

For AD, older adults differed from younger adults in fewer tracts than the other metrics (only 10), and results were mixed (Figure 9). Compared to younger adults, older adults had higher AD in the left arcuate fasciculus, right arcuate fasciculus, left anterior thalamic radiation, right anterior thalamic radiation, right SLF II, left SLF III, and right SLF III, but lower AD in the rostrum of the corpus callosum, splenium of the corpus callosum and left SLF I. Among the models with significant age effects, *R^2^* values ranged from 7% to 19%. AD also differed from the other metrics in that it was the only one to show sex differences. Males exhibited higher AD values than females in two tracts, the corpus callosum body parietal (*β* = −0.31, *p* = 0.002, FDR corrected *p* = 0.002) and temporal (*β* = −0.30, *p* < 0.001, FDR corrected *p* = 0.003) portions (see Supplementary Table 2 for all sex effect statistics).

## 4 Discussion

The present study had two main aims: (1) test whether increasing age has a homogenizing effect on DTI measures of white matter microstructural integrity across tracts, and (2) examine tract-specificity in age and DTI measure associations. In support of the first aim, we examined whether correlations among tract DTI measures differed between younger adults and older adults, under the hypothesis that the effects of aging may result in stronger inter-tract correlations. We found that inter-tract correlations were indeed stronger among older adults than younger adults for FA, RD, and AD, in line with our predictions. Secondly, we extracted a general factor across tract measures in separate models for DTI metrics and age groups. We found that the general factor accounted for greater shared variance among older adults, relative to younger adults. Finally, we assessed how the age groups differed in tract DTI values. For the majority of tracts, older adults had significantly lower FA and higher MD and RD compared to younger adults. Results for AD were mixed, with some tracts showing higher AD for older adults compared to younger adults, and other tracts showing the opposite pattern. Each of these findings will be discussed in detail below.

The present findings suggest that FA, RD, and AD values across tracts are more coherent among older adults compared to younger adults. In comparing inter-tract correlations between age groups, we observed a similar pattern of results for FA, RD, and AD, in which older adults showed stronger inter-tract correlations than younger adults. Mirroring this finding, the general factor for these measures accounted for a larger proportion of variance among older adults compared to younger adults. Previous studies have reported a general factor for FA accounting for 40–50% of shared tract variance within middle-aged and older adult samples (Cox et al., 2016; Gustavson et al., 2019; Penke et al., 2010), in line with the present finding that a general factor for FA captured 48.5% of shared tract variance among older adults. Together, these results support our hypothesis that age has a homogenizing effect across tract DTI measures of white matter microstructural integrity. Our results are consistent with a study by Cox et al. (2016) that found that the proportion of total variance shared across tract FA measures increased with age, in a sample of adults ranging in age from 44 to 77. In contrast, Lövdén et al. (2013) did not find age group differences overall when assessing FA inter-tract correlations between two groups of older adults, a younger (60–72) and older (78–87) cohort. We note, however, that both our study and the one by Cox et al. (2016) compared older adults to much younger individuals; in the present study, younger adults were between the ages of 18 and 37. Taken together, the results of these studies indicate that aging exerts a homogenizing effect on tract FA, RD, and AD measures, that may reach a plateau somewhere in the 6th decade of life.

In contrast to FA, RD, and AD, we did not find compelling evidence of homogenization across tract MD measures with age. While results of the omnibus test showed that the MD correlation matrices were unequal between younger and older adults, relatively few inter-tract correlations differed between the age groups, with some correlations appearing stronger for older adults and others weaker, relative to younger adults. Overall, inter-tract MD correlations appeared to be higher than inter-tract correlations for the other DTI metrics, which is reflected in the finding that a general factor accounted for a high proportion of shared variance (~75%) among tract MD measures for both younger adults and older adults. Given the already high coherence of MD values across tracts in younger adults, it could be that any additional homogenizing impact of age is negligible on MD. This finding requires replication, however, as other studies of middle-aged and older adults have reported lower estimates of shared tract variance accounted for by a general MD factor, ranging from 38% to 62% (Cox et al., 2016; Gustavson et al., 2019; Penke et al., 2010).

While the coherence of MD values across tracts did not vary substantially with age, in contrast to FA and RD, the level of all three of these metrics differed with age. In our age group comparisons, older adults showed lower FA values and higher MD and RD values compared to younger adults in the majority of the examined tracts, consistent with prior studies (Lebel et al., 2012; Storsve et al., 2016; Cox et al., 2016; Schilling et al., 2022; Bennett et al., 2010; Brickman et al., 2012). While there was some variability in the strength of the age effect across tracts, particularly for FA and RD, these results overall suggest that age has a widespread impact on FA, MD and RD values across cerebral white matter. In contrast, age effects on AD values were more selective, and surprisingly inconsistent. For some tracts, younger adults exhibited lower AD values than older adults (ex. anterior thalamic radiations), while for other tracts, younger adults exhibited higher values than older adults (ex. rostrum and splenium of the corpus callosum). While we did not expect to see decreased AD values for older adults relative to younger adults, it should be noted that such findings have been reported previously (Bennett et al., 2010; Burzynska et al., 2010). AD was also unique in that it was the only metric that showed an effect of sex, with males exhibiting higher AD values than females for the parietal and temporal portions of the corpus callosum body. Prior research on sex differences in white matter microstructure has been largely focused on FA and rather mixed, with some studies reporting higher FA values in white matter among females relative to males (Kanaan et al., 2014; Kochunov et al., 2015; Herlin et al., 2024) and others reporting the opposite (Matijevic and Ryan, 2021; Ritchie et al., 2018). This sex difference requires replication, and interpretation should be taken with caution.

The present findings suggest that age-associated alterations to white matter microstructural integrity are widespread. The influence of mechanisms contributing to age-related white matter changes, such as alterations to cardiovascular function (Fuhrmann et al., 2019) and metabolic function (Ryu et al., 2014; van Aalst et al., 2022), is likely not regionally constrained, but instead exerts a strong global impact on white matter integrity. Differential vulnerability to aging across tracts may still arise for multiple reasons, such as elevated sensitivity of “watershed” areas to hypoperfusion as well as varying characteristics of the tracts themselves, like myelin density and axonal composition. Clearly, however, the fact that aging exerts a global impact on white matter integrity must be taken into consideration when exploring potential tract-specific alterations with age, as well as when assessing relationships between specific white matter tracts and various cognitive outcomes. One could conclude that a specific tract is related to a certain cognitive measure, when in truth the cognitive measure is related to cerebral white matter integrity globally. Consistent with this notion, Rabin et al. (2019a) found that tract-specific FA reductions did not predict declines in processing speed over and above global FA reductions, among older adults. Conversely, accounting for global white matter can clarify and strengthen evidence for tract-specificity. For example, when Bennett et al. (2015) investigated the relationship between pattern separation performance and white matter integrity across the adult lifespan (ages 20–89), they observed an association between pattern separation scores and FA values within the fornix, even after controlling for global FA. Accounting for shared variance across tracts may be particularly important when analyzing data from a sample with a wide age range or different age groups, given the present findings that shared variance across tracts differs between younger and older adults.

### 4.1 Limitations and future directions

One caveat to the present results is that this study was limited to group comparisons of younger and older adults. Future studies must additionally examine individuals within middle-age, in order to obtain a more comprehensive understanding of lifespan trajectories of white matter integrity. As well, more recent advancements in diffusion-weighted MRI protocols and modeling, such as NODDI (Zhang et al., 2012) and constrained spherical deconvolution (Tournier et al., 2004; Jeurissen et al., 2014), may provide more nuanced measures of white matter microstructural integrity beyond the conventional diffusion tensor metrics analyzed in this study. Another caveat to the present findings is that the factor analyses of the tract DTI measures were restricted to extracting a single factor. While this decision was motivated by and suited to testing our hypotheses in this study, a one factor solution is not necessarily the optimal factor solution for any of the DTI metrics. Additionally, it might be the case that the optimal factor solution differs between age groups, such that fewer factors explain greater cumulative variance among older adults relative to younger adults. With larger sample sizes that would facilitate modeling of more complex factor structures, future studies could expand on the present work by further characterizing inter-tract relationships and their changes with age. The present results hint at the possibility that age-related “homogenization” of white matter DTI measures plays out differently across tracts; for example, among the FA inter-tract correlations, correlations with the rostrum of the corpus callosum did not differ between age groups to the same extent as correlations with the body segments of the corpus callosum (see Figure 2). We refrain from speculating on these findings here, but they nonetheless illustrate the potential for further investigation in this area.

A major hurdle in studying the effects of age on white matter microstructural health *in-vivo* is the lack of evidence firmly connecting diffusion-weighted MRI-derived estimates of white matter health to age-related pathology. For example, while increased RD among older adults is often interpreted as a sign of reduced myelination, the supporting evidence for this interpretation largely comes from studies of animal models for specific disease states, like the Shiverer mouse model of congenital dysmyelination (Song et al., 2002). There is little evidence, from either human or animal models of typical aging, to indicate that age-related increases in RD actually track levels of demyelination, as confirmed through histopathology. Another point of confusion is the interpretation of AD. Increased AD has been linked to axonal deterioration (Song et al., 2005), but findings of *decreased* AD with age, as seen in present study, are unlikely to indicate axonal damage among the younger adults and/or improved white matter integrity among older adults. Instead, it might be that increased AD among younger adults reflects continuing developmental processes – in line with this, Beck et al. (2021) modeled a u-shaped trajectory for AD across the adult lifespan, with values decreasing from age 18 until around ages 40–50, then increasing. Alternatively, Burzynska et al. (2010) proposed that differing patterns of DTI metric alterations with age (ex. FA decrease/MD increase/RD increase/AD increase vs. FA decrease/RD increase/AD decrease) could reflect distinct underlying pathology (ex. demyelination, Wallerian degeneration, gliosis). In order to strengthen the utility of diffusion-weighted MRI estimates of white matter health for research purposes and, potentially, clinical purposes, further research is needed on the sensitivity and specificity of diffusion-weighted MRI measures as biomarkers of white matter pathology.

## 5 Conclusion

The present cross-sectional comparison of younger adults and older adults provides evidence in support of the notion that age exerts a “homogenizing” influence on cerebral white matter, as we observed greater shared variance across tract DTI measures among older adults, relative to younger adults. This phenomenon presumably stems from the strong global impact of age on white matter integrity, as also evidenced in our findings of widespread age effects across tract DTI measures. Altogether, the results of this study highlight the importance of considering and accounting for global measures when conducting research on white matter, particularly when participants range widely in age.

## Data Availability

The raw data supporting the conclusions of this article will be made available by the authors, without undue reservation.
