# Supplementary material for: Global and tract-specific differences between younger and older adults in DTI measures of white matter integrity
Source: Front Aging Neurosci. 2025 May 30;17:1562660. doi: 10.3389/fnagi.2025.1562660 (PMC12162919; doi:10.3389/fnagi.2025.1562660)
Supplement: Supplementary file 1 [file Table_1.DOCX]

Supplementary Material

|  | FA | | MD | | RD | | AD | |
| --- | --- | --- | --- | --- | --- | --- | --- | --- |
|  | YA | OA | YA | OA | YA | OA | YA | OA |
| CC.R | 0.377 | 0.382 | 0.684 | 0.754 | 0.527 | 0.572 | 0.356 | 0.568 |
| CC.G | 0.317 | 0.457 | 0.714 | 0.732 | 0.511 | 0.57 | 0.446 | 0.638 |
| CC.Bpf | 0.378 | 0.738 | 0.83 | 0.885 | 0.631 | 0.832 | 0.672 | 0.73 |
| CC.Bpm | 0.43 | 0.726 | 0.878 | 0.91 | 0.7 | 0.86 | 0.705 | 0.732 |
| CC.Bc | 0.471 | 0.51 | 0.83 | 0.836 | 0.717 | 0.729 | 0.551 | 0.67 |
| CC.Bp | 0.657 | 0.769 | 0.913 | 0.909 | 0.821 | 0.886 | 0.8 | 0.731 |
| CC.Bt | 0.807 | 0.842 | 0.932 | 0.95 | 0.91 | 0.927 | 0.81 | 0.845 |
| CC.S | 0.481 | 0.514 | 0.79 | 0.842 | 0.638 | 0.707 | 0.407 | 0.504 |
| L.AF | 0.831 | 0.801 | 0.973 | 0.953 | 0.957 | 0.93 | 0.803 | 0.855 |
| R.AF | 0.827 | 0.828 | 0.972 | 0.971 | 0.952 | 0.965 | 0.812 | 0.848 |
| L.AR | 0.688 | 0.709 | 0.784 | 0.724 | 0.808 | 0.756 | 0.537 | 0.557 |
| R.AR | 0.493 | 0.752 | 0.801 | 0.777 | 0.758 | 0.811 | 0.608 | 0.519 |
| L.ATR | 0.173 | 0.625 | 0.818 | 0.784 | 0.591 | 0.703 | 0.515 | 0.726 |
| R.ATR | 0.063 | 0.466 | 0.812 | 0.754 | 0.557 | 0.632 | 0.52 | 0.739 |
| L.CBD | 0.548 | 0.661 | 0.922 | 0.931 | 0.798 | 0.867 | 0.571 | 0.561 |
| R.CBD | 0.361 | 0.675 | 0.941 | 0.949 | 0.709 | 0.881 | 0.567 | 0.641 |
| L.CBV | 0.272 | 0.632 | 0.759 | 0.769 | 0.676 | 0.785 | 0.481 | 0.498 |
| R.CBV | 0.421 | 0.604 | 0.787 | 0.745 | 0.714 | 0.765 | 0.484 | 0.48 |
| L.EMC | 0.419 | 0.69 | 0.805 | 0.846 | 0.64 | 0.811 | 0.532 | 0.62 |
| R.EMC | 0.471 | 0.689 | 0.805 | 0.835 | 0.731 | 0.806 | 0.414 | 0.614 |
| L.FAT | 0.66 | 0.747 | 0.916 | 0.926 | 0.838 | 0.881 | 0.669 | 0.785 |
| R.FAT | 0.598 | 0.769 | 0.911 | 0.941 | 0.772 | 0.905 | 0.653 | 0.757 |
| L.ILF | 0.694 | 0.71 | 0.839 | 0.834 | 0.837 | 0.851 | 0.622 | 0.524 |
| R.ILF | 0.658 | 0.66 | 0.87 | 0.839 | 0.836 | 0.852 | 0.589 | 0.529 |
| L.MLF | 0.758 | 0.836 | 0.905 | 0.876 | 0.883 | 0.897 | 0.71 | 0.704 |
| R.MLF | 0.784 | 0.847 | 0.918 | 0.897 | 0.915 | 0.922 | 0.774 | 0.726 |
| L.OR | 0.585 | 0.674 | 0.807 | 0.854 | 0.806 | 0.86 | 0.611 | 0.584 |
| R.OR | 0.75 | 0.634 | 0.831 | 0.801 | 0.877 | 0.802 | 0.607 | 0.589 |
| L.SLFI | 0.497 | 0.668 | 0.892 | 0.933 | 0.694 | 0.854 | 0.651 | 0.743 |
| R.SLFI | 0.667 | 0.616 | 0.922 | 0.935 | 0.823 | 0.849 | 0.594 | 0.737 |
| L.SLFII | 0.762 | 0.743 | 0.957 | 0.943 | 0.925 | 0.907 | 0.795 | 0.874 |
| R.SLFII | 0.769 | 0.811 | 0.966 | 0.956 | 0.927 | 0.94 | 0.802 | 0.882 |
| L.SLFIII | 0.774 | 0.726 | 0.962 | 0.916 | 0.921 | 0.874 | 0.797 | 0.826 |
| R.SLFIII | 0.741 | 0.833 | 0.946 | 0.935 | 0.894 | 0.928 | 0.738 | 0.833 |
| L.UF | 0.421 | 0.699 | 0.797 | 0.841 | 0.677 | 0.828 | 0.622 | 0.638 |
| R.UF | 0.578 | 0.701 | 0.79 | 0.846 | 0.782 | 0.804 | 0.464 | 0.707 |

Supplementary Table 1. Loadings from the factor analyses per age group and DTI metric.


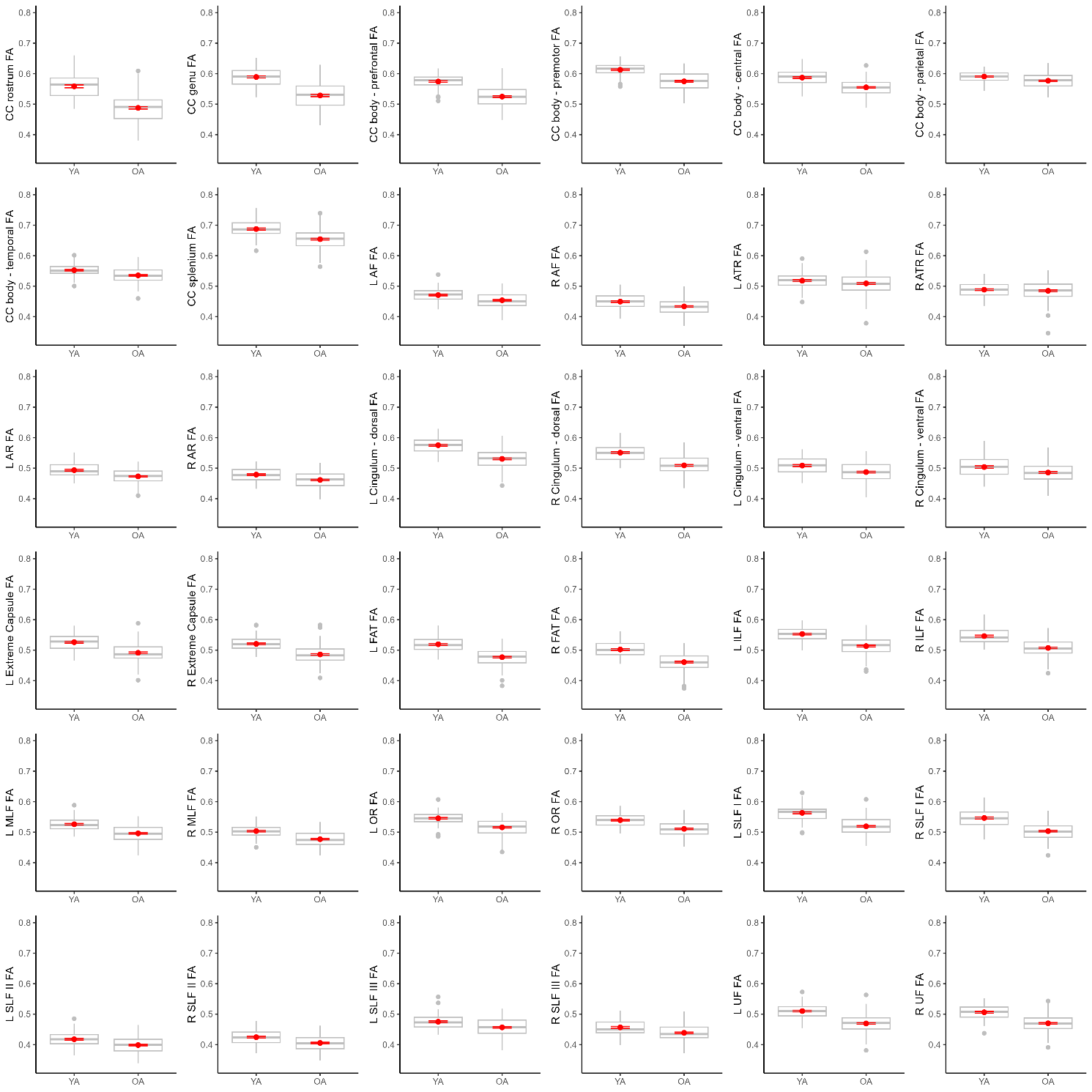


Supplementary Figure 1. Boxplots showing differences in tract FA values between younger adults (YA) and older adults (OA). Red dots represent the means, red error bars represent the standard error (± 1 SE), and gray dots are outliers (values 1.5 times the interquartile range over the third quartile or under the first quartile).


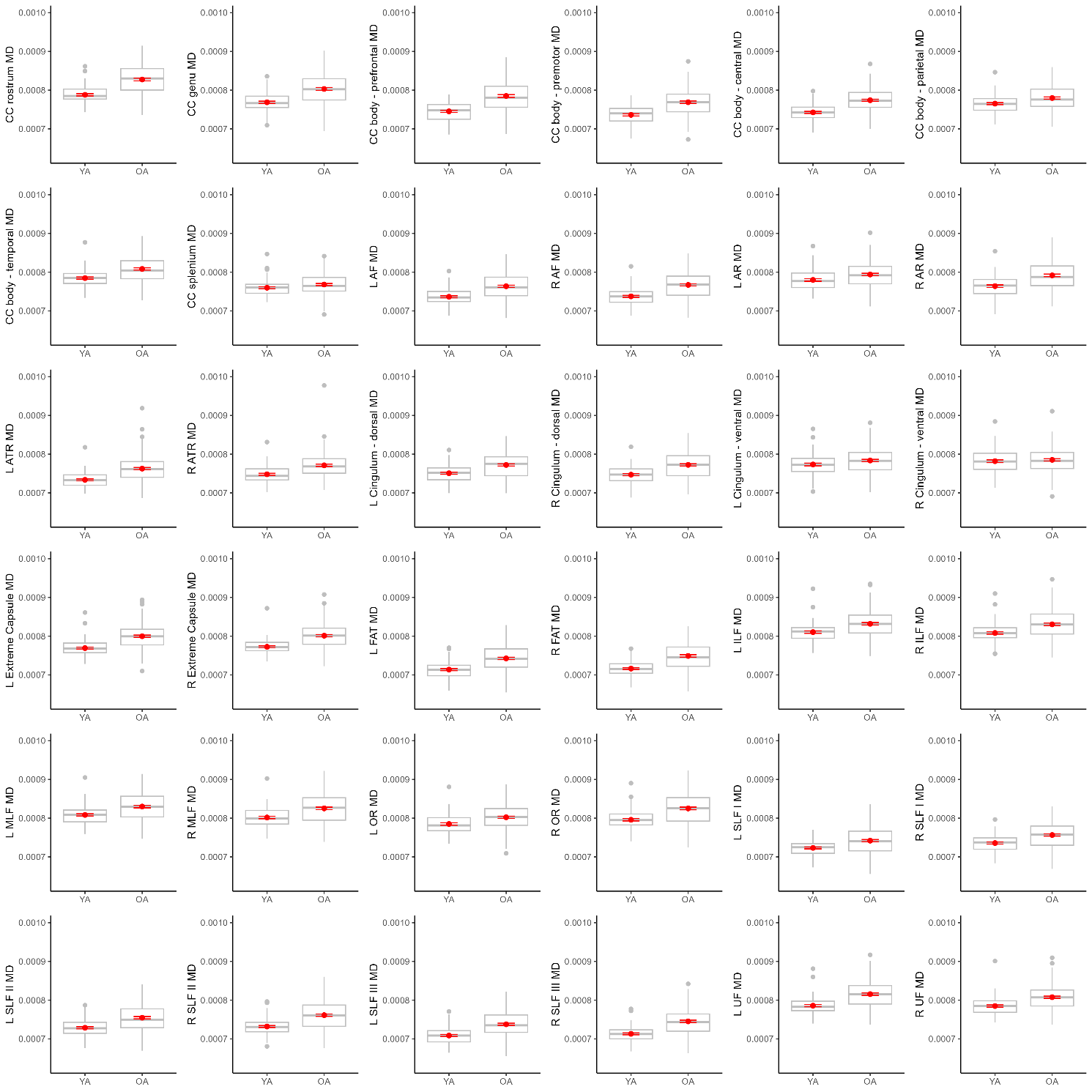


Supplementary Figure 2. Boxplots showing differences in tract MD values between younger adults (YA) and older adults (OA). Red dots represent the means, red error bars represent the standard error (± 1 SE), and gray dots are outliers (values 1.5 times the interquartile range over the third quartile or under the first quartile).


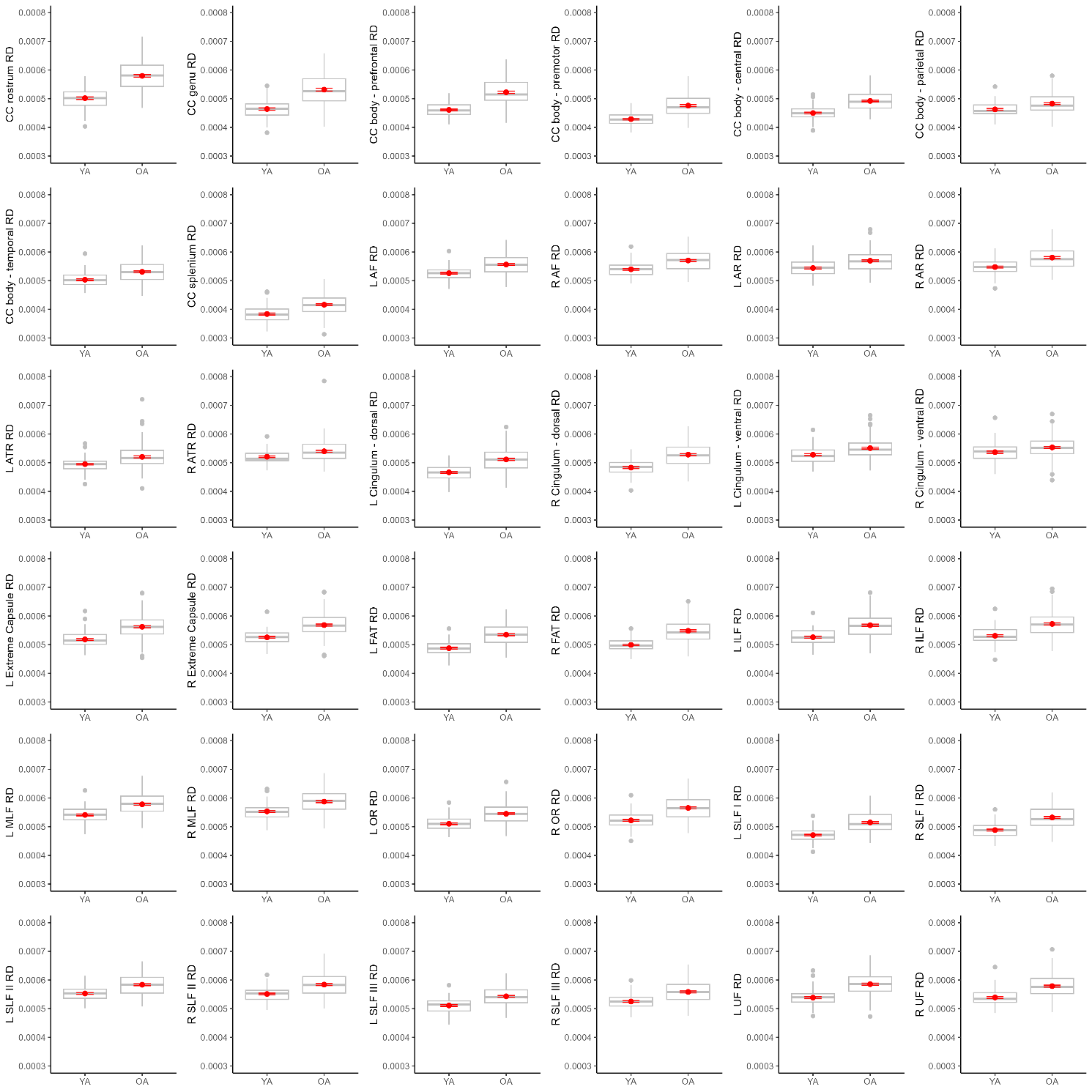


Supplementary Figure 3. Boxplots showing differences in tract RD values between younger adults (YA) and older adults (OA). Red dots represent the means, red error bars represent the standard error (± 1 SE), and gray dots are outliers (values 1.5 times the interquartile range over the third quartile or under the first quartile).


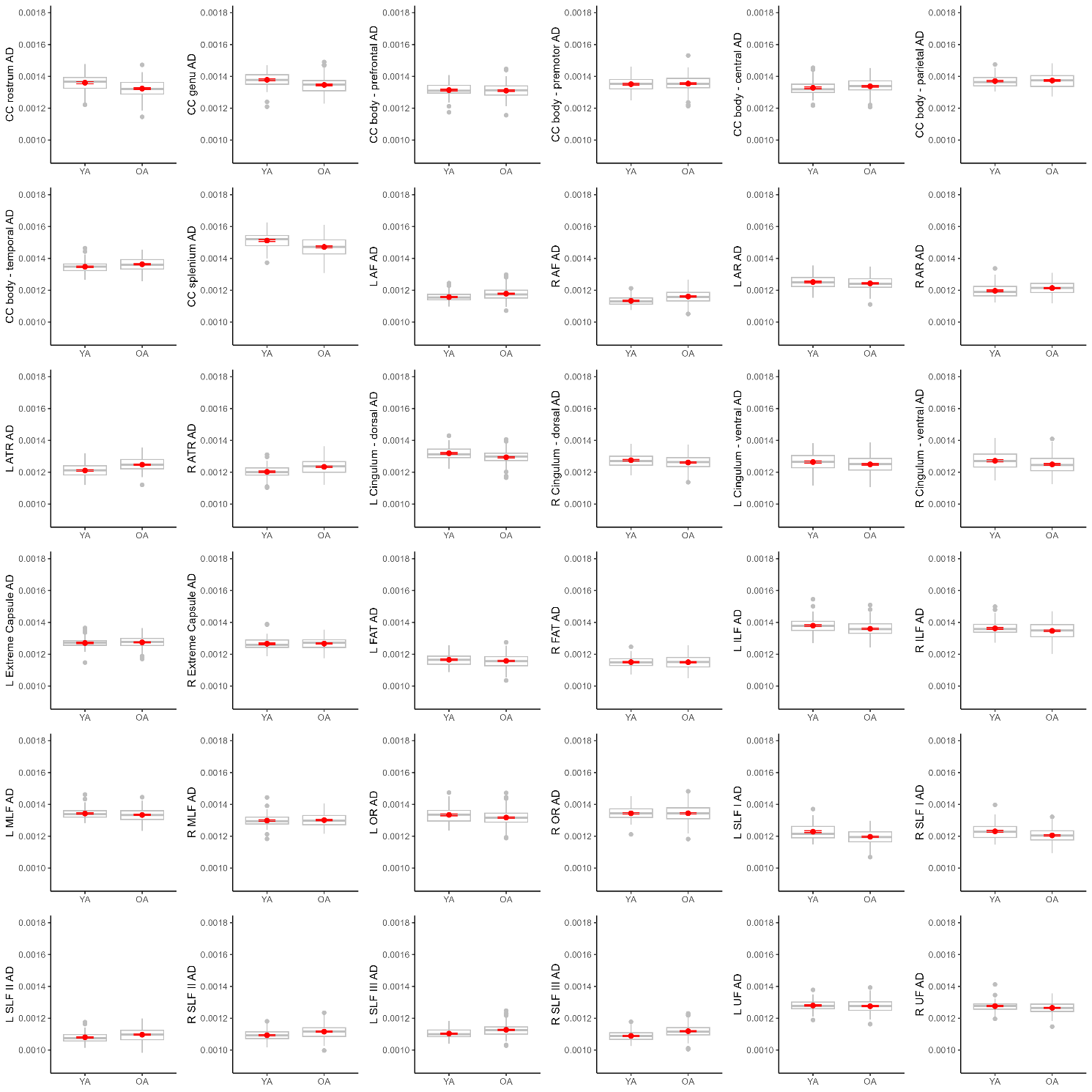


Supplementary Figure 4. Boxplots showing differences in tract AD values between younger adults (YA) and older adults (OA). Red dots represent the means, red error bars represent the standard error (± 1 SE), and gray dots are outliers (values 1.5 times the interquartile range over the third quartile or under the first quartile).

|  | **FA** | | **MD** | | **RD** | | **AD** | |
| --- | --- | --- | --- | --- | --- | --- | --- | --- |
| **Tract** | ***β*** | ***Adj R²*** | ***β*** | ***Adj R²*** | ***β*** | ***Adj R²*** | ***β*** | ***Adj R²*** |
| CC.R | -0.05 | 0.43 | -0.03 | 0.23 | 0.02 | 0.41 | -0.11 | 0.11 |
| CC.G | -0.09 | 0.39 | 0 | 0.16 | 0.06 | 0.33 | -0.12 | 0.16 |
| CC.Bpf | -0.04 | 0.40 | -0.01 | 0.24 | 0.01 | 0.35 | -0.04 | 0.04 |
| CC.Bpm | 0.01 | 0.33 | -0.06 | 0.22 | -0.04 | 0.32 | -0.07 | 0.02 |
| CC.Bc | -0.05 | 0.27 | -0.15 | 0.27 | -0.05 | 0.34 | -0.2 | 0.05 |
| CC.Bp | -0.03 | 0.07 | -0.2 | 0.08 | -0.08 | 0.08 | -0.31** | 0.09 |
| CC.Bt | -0.06 | 0.10 | -0.16 | 0.14 | -0.05 | 0.13 | -0.3** | 0.11 |
| CC.S | 0.18 | 0.23 | -0.16 | 0.04 | -0.2 | 0.21 | 0.03 | 0.08 |
| L.AF | -0.11 | 0.10 | -0.05 | 0.17 | 0.02 | 0.17 | -0.16 | 0.09 |
| R.AF | -0.04 | 0.07 | -0.09 | 0.18 | -0.04 | 0.16 | -0.16 | 0.12 |
| L.AR | 0.07 | 0.15 | -0.1 | 0.04 | -0.11 | 0.12 | -0.04 | 0.00 |
| R.AR | -0.04 | 0.09 | -0.06 | 0.13 | -0.03 | 0.16 | -0.09 | 0.04 |
| L.ATR | -0.13 | 0.05 | -0.01 | 0.18 | 0.06 | 0.10 | -0.14 | 0.19 |
| R.ATR | -0.11 | 0.02 | -0.11 | 0.12 | -0.01 | 0.06 | -0.21 | 0.13 |
| L.CBD | -0.05 | 0.34 | -0.03 | 0.12 | 0.02 | 0.27 | -0.1 | 0.12 |
| R.CBD | -0.02 | 0.30 | -0.06 | 0.15 | -0.03 | 0.27 | -0.08 | 0.05 |
| L.CBV | 0.06 | 0.09 | -0.18 | 0.05 | -0.16 | 0.11 | -0.12 | 0.02 |
| R.CBV | 0.03 | 0.05 | -0.24 | 0.05 | -0.17 | 0.06 | -0.2 | 0.07 |
| L.EMC | -0.05 | 0.25 | -0.02 | 0.19 | 0.01 | 0.24 | -0.09 | 0.01 |
| R.EMC | 0.06 | 0.26 | -0.13 | 0.19 | -0.11 | 0.26 | -0.11 | 0.02 |
| L.FAT | -0.05 | 0.35 | 0.05 | 0.18 | 0.05 | 0.31 | 0.02 | 0.00 |
| R.FAT | 0.07 | 0.33 | -0.01 | 0.21 | -0.05 | 0.32 | 0.09 | 0.00 |
| L.ILF | 0.11 | 0.32 | -0.14 | 0.09 | -0.14 | 0.23 | -0.07 | 0.03 |
| R.ILF | 0.16 | 0.34 | -0.13 | 0.09 | -0.15 | 0.23 | -0.03 | 0.01 |
| L.MLF | 0.04 | 0.25 | -0.07 | 0.09 | -0.07 | 0.20 | -0.04 | 0.00 |
| R.MLF | -0.01 | 0.22 | -0.12 | 0.11 | -0.07 | 0.17 | -0.18 | 0.02 |
| L.OR | -0.01 | 0.26 | 0.02 | 0.06 | 0.01 | 0.22 | 0.02 | 0.01 |
| R.OR | 0.14 | 0.26 | -0.04 | 0.13 | -0.1 | 0.25 | 0.07 | -0.01 |
| L.SLFI | -0.03 | 0.36 | -0.05 | 0.10 | -0.01 | 0.30 | -0.07 | 0.12 |
| R.SLFI | 0.09 | 0.36 | -0.07 | 0.12 | -0.11 | 0.30 | 0.04 | 0.08 |
| L.SLFII | -0.09 | 0.12 | -0.03 | 0.14 | 0.02 | 0.16 | -0.11 | 0.04 |
| R.SLFII | 0 | 0.11 | -0.07 | 0.17 | -0.04 | 0.18 | -0.11 | 0.07 |
| L.SLFIII | -0.08 | 0.11 | -0.05 | 0.20 | 0.01 | 0.20 | -0.13 | 0.10 |
| R.SLFIII | -0.07 | 0.09 | -0.07 | 0.20 | -0.01 | 0.18 | -0.17 | 0.15 |
| L.UF | -0.04 | 0.35 | -0.09 | 0.17 | -0.04 | 0.27 | -0.16 | 0.06 |
| R.UF | 0.03 | 0.29 | -0.12 | 0.13 | -0.08 | 0.23 | -0.13 | 0.02 |

Supplementary Table 2. Results of regressions testing for age group differences in tract DTI metrics. Sex effect β’s are shown next to the adjusted R² for the model. FDR-corrected p-values are represented as follows: *** p < 0.001, ** p < 0.01, * p < 0.05
